# 

**Published:** 1844-02

**Authors:** 


### CONTENTS

OF NO. II.

---

### ORIGINAL COMMUNICATIONS.

### ESSAYS AND CASES.

ART. I.—On a Ferruginous preparation, termed, vulgarly, "Clinkers," in certain Diseases. By W. S. McMURTRY, M.D., of Benton, Mississippi. - - 97

ART. II.—Remarks on the Diseases which prevail in Tennessee during Summer and Winter. By JOHN W. RICHARDSON, M.D., of Rutherford county, Tenn. 105

ART. III.—A Case of Renal Calculi, with abscess and destruction of the right kidney. Reported by R. MARTIN, M.D., of Nashville, Tenn. - - - - 119

ART. IV.—A Case of protracted and difficult Labour, followed by Sloughing of the Vagina, and Recto-Vaginal Septum. By Dr. JOHN P. ANDERSON, of Sparta, Alabama. - - - - - - - - - 121

### REVIEWS.

ART. V.—A Treatise on Food and Diet: with observations on the Dietetical Regimen, suited for Disordered States of the Digestive Organs; and an account of the Dietaries of some of the principal Metropolitan and other Establishments for Paupers, Lunatics, Criminals, &c. By JONATHAN PEREIRA, M.D., F.R.S. & L.S., &c. Edited by CHARLES A. LEE, M.D. New York: J. & H. G. Langley. 1vol. 8vo. pp. 325. 1843. 123

ART. VI.—Principles of Medicine: comprising General Pathology and Therapeutics, and a brief general view of Etiology, Nosology, Semeiology, Diagnosis and Prognosis. By CHARLES J. B. WILLIAMS, M.D. F.R.S. &c. With Additions and Notes. By MEREDITH CLYMER, M.D., &c. Philadelphia: Lea & Blanchard. 1844. 8vo. pp. 383. - - 144

SELECTIONS FROM AMERICAN AND FOREIGN JOURNALS.

On the Effects of Opium on the Infant Subject. - - 157
On the Influence of Opium upon the Catamenial Functions. 164
Dr. Gregory on Small-Pox. - - - - - - - 166
Vital Statistics of Sheffield. - - - - - - - 171
Poverty of Medical Men: how to eke out a livelihood. - 174
Professional Success.—Gravity *vs.* Hilarity. - - - 175
Dipsopathia—the latest Medical Humbug. - - - - 176
Antagonism of Phthesis and Intermittent Fever. - - 176
Toxicological effects of Sulphate of Quinine. - - - 177
Milk injected into the Veins. - - - - - - - - 177
Purification of the air without renewing it. - - - 178
Fair at the New York State Lunatic Asylum. - - 178
Mortality in Boston in 1843. - - - - - - - 179
Castration performed on the patient by himself. - - 179
Treatment of Hooping Cough by Cochineal. - - - 180

ORIGINAL INTELLIGENCE.

Jarvis on Insanity. - - - - - - - - 181
A New Medical Journal. - - - - - - - 183
Nashville Medical School. - - - - - - - 183
Professor Litton's Introductory. - - - - - - 183
Medical Ethics. - - - - - - - - - 184
Medical College of Louisiana. - - - - - - 184
Rush Medical College, Chicago. - - - - - - 185
New Life-Preserver. - - - - - - - - 186
Medical Classes. - - - - - - - - - 187
Mesmerism. - - - - - - - - - - 187
Medical Institute of Louisville. - - - - - - 187
Western Journal. - - - - - - - - 188

